# Calibration and Improvement of an Odometry Model with Dynamic Wheel and Lateral Dynamics Integration

**DOI:** 10.3390/s21020337

**Published:** 2021-01-06

**Authors:** Máté Fazekas, Péter Gáspár, Balázs Németh

**Affiliations:** 1 Department of Control for Transportation and Vehicle Systems, Budapest University of Technology and Economics, H-1111 Budapest, Hungary; 2 Systems and Control Laboratory, Institute for Computer Science and Control (SZTAKI), H-1111 Budapest, Hungary; peter.gaspar@sztaki.hu (P.G.); balazs.nemeth@sztaki.hu (B.N.)

**Keywords:** positioning, wheel odometry, calibration, sensor fusion, Gauss–Newton regression, Kalman-filtering

## Abstract

Localization is a key part of an autonomous system, such as a self-driving car. The main sensor for the task is the GNSS, however its limitations can be eliminated only by integrating other methods, for example wheel odometry, which requires a well-calibrated model. This paper proposes a novel wheel odometry model and its calibration. The parameters of the nonlinear dynamic system are estimated with Gauss–Newton regression. Due to only automotive-grade sensors are applied to reach a cost-effective system, the measurement uncertainty highly corrupts the estimation accuracy. The problem is handled with a unique Kalman-filter addition to the iterative loop. The experimental results illustrate that without the proposed improvements, in particular the dynamic wheel assumption and integrated filtering, the model cannot be calibrated precisely. With the well-calibrated odometry, the localization accuracy improves significantly and the system can be used as a cost-effective motion estimation sensor in autonomous functions.

## 1. Introduction

Autonomous robots, including self-driving vehicles, operate in the see-think-act cycle [1]. In general, the algorithms have four main layers: environment perception, state estimation, decision making and trajectory planning, and motion control. The first two are critical because their errors can result in misbehavior regardless of if the last two layers are well-developed. The state estimation is responsible for the determination of necessary quantities, e.g., accelerations, yaw rate, or the pose (position and orientation) of the vehicle. For the process, three contradictory requirement concerns: high accuracy, robustness for different conditions and the application of cost-effective sensors. The main localization system for outdoor ground autonomous vehicles is the Global Navigation Satellite System (GNSS) sensor, however, the accuracy and sampling rate of the the cost-effective automotive grade type are not enough for autonomous driving functions [2]. Hence, other methods have to be integrated into the estimation as well. This paper focuses on the modeling and calibration of the wheel odometry model with dynamic assumptions applied to self-driving cars for the localization tasks.

Odometry is the use of data from motion sensors to estimate change in pose over time. Several methods exist, such as inertial, visual, laser or wheel odometry, and any of the methods can be applied in a multisensor fusion algorithm, e.g., visual-inertial odometry. A detailed survey about the advantages and disadvantages of the methods can be found in ref. [3], here only a short summary is presented. The disadvantage of the Light Detection and Ranging (LiDAR) based visual odometry is the requirement of expensive sensor. The inertial odometry relies on the Inertial Measurement Unit (IMU), where the bias and noise of the accelerometers and gyroscopes are significant, and may results in faulty localization [4]. The mono camera-based visual odometry estimates the motion only up to a scale, and the lack of features and non-overlapping images are also a problem [5]. These disadvantages are compensated in the visual-inertial odometry, a state-of-the-art method called ROVIO can be found in ref. [6]. However, in the case of a car it requires improvement as it is mentioned in ref. [7], where the ROVIO is supplemented by a wheel encoder-based method to make the estimation reliable. The problem is the error-prone IMU measurements in the self-driving application, where strong and frequent accelerations are lacked, thus the signal-to-noise-ration is low. A similar problem occurs in the well-known GNSS/IMU fusion algorithm, where the method should estimate the pose of the vehicle and the IMU biases in parallel. The estimation is poor, when the GNSS signal is weak, and becomes highly erroneous in GNSS outages. In ref. [4], the problem is solved by integrating the wheel motion, which clearly improves the results.

The wheel odometry relies on the measurements of the wheel encoder sensors, which are equipped on the vehicles due to the necessity of the Anti-Lock Braking System (ABS), thus the method is the most cost-effective localization technique. The previous papers ([4,7]) demonstrate that this type of odometry can improve other methods. Moreover, there are special cases where the wheel odometry should be the main localization algorithm, such as driving in weak lighting conditions or in environments without clear features, low-speed maneuvering, and in parking scenarios [8]. The wheel slipping can result in uncertain behavior, but in normal driving and weather conditions this effect is negligible and also detection algorithms exist, see [9]. The errors in the road surface, such as bumps, ruts, and potholes can corrupt the estimation as well, but these are only temporary faults and can be detected by functions [10]. The main disadvantage of the encoder-based method is the effect of parameter uncertainty because a precise model is required to the pose estimation. The geometry parameters of the model, such as wheel circumference or track width can be calibrated during installation, however it concern only for the actual tire and its condition. Due to wear, different loads or tire change the re-calibration is needful. Furthermore, non-static behavior, such as dynamic circumference change in the bends can not be calibrated at the static installation, thus these effects should be learned from data while driving.

Finally, a precisely calibrated wheel odometry model would have further advantages than the mentioned localization problems, e.g., accurate bias and noise estimation for GNSS and IMU sensors or plausibility check for other quantities. It is clear that the fusion of different localization method needful in an autonomous vehicle [11], but it is also important that the individual methods perform at its own maximum performance. Therefore, the wheel odometry model has a clear and significant role in the autonomous driving technology, however, a precise and well-calibrated model is difficult to obtain.

### 1.1. Related Works of Wheel-Encoder Based Odometry

The estimates with respect to a dynamic system have two possible aims. One is when the states are estimated or filtered (tracking), and the other is when the goal is to estimate the system parameters (calibration). However, there are methods that deal with the joint problem of tracking and calibration. It can be performed with several methods, such as a Switching Kalman-filter, which extends the filtering behavior of the filter with a switching approach represented by system parameters which are also estimated, see [12]. For the joint estimation problem the Monte Carlo methods can be applied also [13], such as the particle filter [14], or the SMC which is a sequential parameter and state estimation technique, see [15]. Nevertheless, the main focus in this paper is on the calibration of the wheel-odometry model, thus in the following the parameter estimation techniques are discussed in detail.

This paper deals with real-sized vehicles, however the works in the mobile robot field are summarized first because the wheel encoder-based odometry is a widely applied method in these applications. One of the earliest paper which deals with the odometry and its errors is a map-making example in [16]. The field of the applicable odometry models for mobile robots is a well-explored area, an odometry and error model of a differential drive robot can be found in ref. [17], in ref. [18] the model also make assumptions on wheel longitudinal slips, and a model for wheeled mobile robots which incorporating linear acceleration is examined in ref. [19].

The calibration problem of the applied odometry models appears parallel with the usage of the wheel encoder-based techniques [20]. The first methods operates with pre-programmed paths and requires human interactions [17,21]. The odometry error can be handled in two different ways. One is when the error is compensated with additive components, for example in the input of the model [22]. In ref. [23] the method examined in detail focusing on distance dependence of the drift, which turned out to be quadratic, because the angular velocity error is integrated twice. Other additive component can be a varying covariance matrix of the model [24]. This is a fully general approach with the advantage of the capability to adapt rapidly to every type of error source, i.e., unmodeled dynamics, disturbances or noise in the input of the odometry model. However, measurements of a drift-less sensor are required and it is difficult to determine how could the raw odometry perform alone.

The other way to handle the odometry error is based on the assumption that the drift is a consequence of parameter uncertainty. The parameter estimation can be performed parallel with the state filtering, where the unknown quantities are handled as state variables. Most often, the Augmented Kalman-filter is used [25]. In the filter, the wheel odometry is fused with other sensor measurements, i.e., gyroscope [26], Differential Global Positioning System (DGPS) [27], camera [25] or laser range finder [28]. The parameters are calibrated online in a simple and automatic way in every time step, which is the main advantage of the method, but several limitations exist. Without further examination of the actual inputs of the odometry model, observability issues may appear [29], and it is difficult to guarantee the convergence to the optimal parameter values [30], and the effect of the measurement noise is significant due to the possible change of the parameter values in every time step [31].

The other and more common way to handle the parameter estimation is as a regression problem. The disadvantage is that the wheel odometry model is nonlinear which results in a non-convex optimization problem, which is much more difficult to solve. The odometry model is separated into two parts and the estimation is executed in two linear fitting steps in [32], but this increases the effect of noise and easily results in biased parameter estimation. The method is improved with a maximum likelihood estimation in ref. [31], and also upgraded in ref. [33] with the addition of an iterative loop. In [34] the general nonlinear least square problem is handled with the Gauss–Newton approximation and the observability of the calibration parameters is also taken into account. The nonlinear estimation problem is handled with a linearized system dynamics and integrated prediction error minimization in ref. [18].

All of the previously mentioned works deal with mobile robots with differential drive. The wheel-encoder based odometry appeared in the automotive industry as part of the parking assist system for the localization task [35]. The applicable models for car-like vehicle localization is examined in several papers. In ref. [36] a comparison of the rear and front axle models for parking is presented, and it is also shown that the front suspension system should be taken into account in the localization model, because of the effect of nonlinearities is significant in real-sized passenger cars compared to small robots. A detailed comparison of the different steering geometries with the scope of positioning error can be found in ref. [37], and the four-wheel-steering odometry model with a linearized state space is presented in ref. [38]. Other works deal with non-systematic odometry errors, such as wheel slipping for small exploratory rover [39], and in the case of parking on slippery terrain [9].

In the field of car-like vehicles, much less papers were presented for the calibration problem. A basic parameter estimation algorithm can be found in ref. [40], where the outdoor mobile robot is driven on straight and circular trajectories, and the path is measured with a high-precision positioning system. ref. [41] uses a radio-controlled (RC) small-scaled car and investigates the resulted in paths with uncalibrated wheel diameter and track width on a pre-programmed closed oval route. In ref. [42] the same robot and path are applied, but the experimental orientation error is utilized at the end of the path instead of the position error to eliminate the small-angle approximation. The four-wheel-steering of small RC cars is calibrated with a sequential method in ref. [38] operating with straight driving with various parallel steering.

Only a few works deal with the wheel encoder-based odometry calibration for real-sized vehicles. In ref. [43] the previously mentioned Augmented Kalman-filter is applied to estimate the parameters of the rear track from an onboard GNSS sensor. The whole system is cost-effective, but it was mentioned that there are several limitations. The same parameters are identified in ref. [44] based on extremely expensive sensors, such as a real-time kinematic GNSS and fiber-optic gyroscopes. In ref. [45] the odometry model is improved with a neural network, which is trained to determine the displacement and rotation of the car from the wheel rotation inputs. A similar idea is presented in ref. [46], where the error between the measurements and the output of the odomery model with nominal parameters is estimated with a Gaussian process, which uses up the result of the nominal odometry model as an input. The disadvantage of these networks and processes is the requirement of a huge data set to build a general model, and also it is difficult to validate the resulting function.

### 1.2. Contributions and Organization of the Paper

As we can see, there are plenty of works on the topic of small mobile robots with differential drive, and car-like steering and drive. However, these models neglect several effects that have a high impact on the localization of real-size vehicles in contrast with the small robots. For example, the wheel slip has less impact, in normal driving conditions, because of the heavy weight, but the influence of sideslip is much higher due to the greater forces [47]. Furthermore, in small robots, the wheel is often made from plastic or does not contain inner liner, thus the circumference is assumed to be constant. In ref. [45], it is shown that the constant assumption is not proper, and also in our previous work it is presented that the effect of dynamic change of the circumference in bends has high impact [48]. The main contribution of this paper is a novel wheel odometry model, in which the sideslip and dynamic wheel model are included. To the best of our knowledge, this is the first published paper where these effects are taken into account in wheel encoder-based localization and odometry model calibration. The model and two motivation example can be found in Section 2.1.

The continuous estimation of sideslip is difficult with cost-effective sensors, and maybe this is why it is neglected generally. Therefore, our next contribution in Section 3 is a continuous sideslip estimation algorithm, which operates with automotive grade type onboard sensors, such as GNSS, IMU, and wheel and steering encoder. The method is not real-time, nevertheless it is sufficient for the odometry model calibration.

Section 4 a calibration method, which is the last contribution of the paper. The algorithm operates in a general case: it does not require any pre-programmed path, it is automatic, and it only signals of onboard automotive-grade type sensors are used while the vehicle travels. The unique addition of the technique to the well-known least square-based estimation is a Kalman-filter, with which the negative effects of the noisy measurements can be decreased thereby increasing the accuracy of the calibration.

Our test vehicle and the applied measurement introduced in Section 5. The validity of the proposed model and calibration approach are demonstrated in Section 6 through a real experiment, and the calibrated odometry model as a motion estimation sensor is examined also. Finally, the paper is concluded in Section 7.

## 2. Vehicle Model and Motivation Examples

### 2.1. Novel Wheel Odometry Model with Dynamic Assumptions

**Figure 1:**
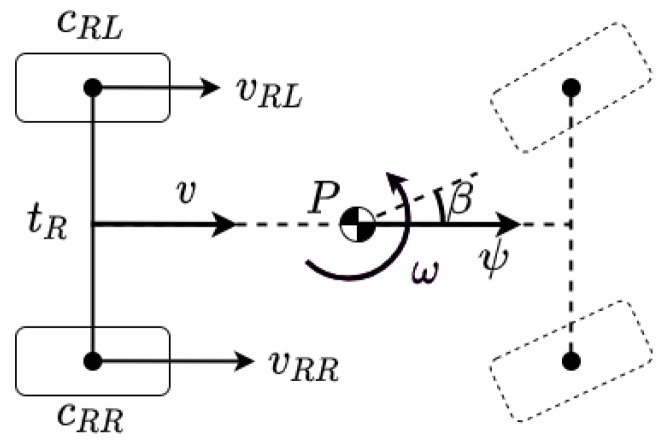
Two-wheel odometry model.

The dead-reckoning navigation is based on a model, where state vector $x_{k}$ contains the longitudinal and lateral vehicle positions $p_{x,k},p_{y,k}$ of the reference point *P*, which is the center of gravity in our model, and the heading angle $\psi_{k}$, as it is illustrated in Figure 1. The inputs of the model are the measured number of wheel rotation revolutions per second $n_{i}$, and the $\beta_{k-1}$ which is the sideslip of the vehicle.

The pose change of the vehicle is based on the longitudinal $v_{k-1}$ and angular $\omega_{k-1}$ velocities and calculated as

(1) $$\Delta p_{x,k}=v_{k-1}\cdot \cos\left(\psi_{k-1}+\frac{\omega_{k-1}}{2}+\beta_{k-1}\right),$$

(2) $$\Delta p_{y,k}=v_{k-1}\cdot \sin\left(\psi_{k-1}+\frac{\omega_{k-1}}{2}+\beta_{k-1}\right),$$

(3) $$\Delta \psi_{k}=\omega_{k-1}.$$

The velocities are computed utilizing the rear wheel rotations in case of a two-wheel model-based odometry. Experimental tests demonstrated that in normal driving and weather conditions the wheel slip can be negligible, consequently, the velocities are calculated as follows

(4) $$v_{k}=\left(n_{RL,k}\cdot c_{RL,k}+n_{RR,k}\cdot c_{RR,k}\right)/2,$$

(5) $$\omega_{k}=\left(n_{RR,k}\cdot c_{RR,k}-n_{RL,k}\cdot c_{RL,k}\right)/t_{R},$$

where $c_{i,k}=2\pi r_{i,k}$ is the actual wheel circumference, $t_{R}$ is the rear track. $r_{i,k}$ is the actual rolling radius. The slight change of the wheel radius due the effect of vertical dynamic is generally neglected, because the odometry based localization is widely used in low speed circumstances i.e., automated parking. However, the sensor measurements used for calibration are collected from normal city and suburb driving, where the dynamic is certainly higher. Therefore, the slight change due to the vertical load transfer should be considered. Accordingly, the current wheel circumferences used in our model are defined as

(6) $$c_{RL,k}=c_{e,RL}+D\cdot a_{y,k},$$

(7) $$c_{RR,k}=c_{e,RR}-D\cdot a_{y,k}=c_{e,RL}+c_{d}-D\cdot a_{y,k},$$

where the $c_{e,i}$ is the effective wheel circumference, $c_{d}$ is the difference between the effective values, $a_{y,k}$ is the lateral acceleration and *D* is a parameter that takes into account the effect of vertical dynamics and will be described as load transfer coefficient.

### 2.2. System Model for the Calibration

The presented odometry model results in a nonlinear state-space representation, such as

(8) $$x_{k}=f\left(x_{k-1},u_{k-1},\theta \right).$$

The state vector $x_{k}$ contains the pose values, the input vector $u_{k-1}$ is formulated by the wheel rotations, the sideslip, and the lateral acceleration, and due to every state is measured, in the observation equation the output $y_{k}$ is equal with the state $x_{k}$,

(9) $$x_{k}={\left[p_{x,k},p_{y,k},\psi_{k}\right]}^{T},\;u_{k-1}={\left[n_{RL,k-1},n_{RR,k-1},a_{y,k-1},\beta_{k-1}\right]}^{T},\;y_{k}=x_{k}.$$

The vehicle model parameters are arranged in the parameter vector θ,

(10) $$\theta =[c_{e,RL},c_{d},t_{R},D],$$

and the velocities are calculated with these in the following way,

(11) $$v_{k}=c_{e,RL}\frac{n_{RL,k}+n_{RR,k}}{2}+c_{d}\frac{n_{RR,k}}{2}+D\frac{\left(n_{RL,k}-n_{RR,k}\right)\cdot a_{y,k-1}}{2},$$

(12) $$\omega_{k}=c_{e,RL}\frac{n_{RR,k}-n_{RL,k}}{t_{R}}+c_{d}\frac{n_{RR,k}}{t_{R}}+D\frac{-\left(n_{RL,k}+n_{RR,k}\right)\cdot a_{y,k-1}}{t_{R}}.$$

The state transition equations are based on these velocities through the presented two-wheel odometry model, such as

(13) $$\underset{x_{k}}{\underbrace{\left[\begin{array}{c} p_{x,k}\\ p_{y,k}\\ \psi_{k} \end{array}\right]}}=\underset{f}{\underbrace{\left[\begin{array}{c} p_{x,k-1}+v_{k-1}\cdot \cos\left(\psi_{k-1}+\frac{\omega_{k-1}}{2}+\beta_{k-1}\right)\\ p_{y,k-1}+v_{k-1}\cdot \sin\left(\psi_{k-1}+\frac{\omega_{k-1}}{2}+\beta_{k-1}\right)\\ \psi_{k-1}+\omega_{k-1} \end{array}\right]}}.$$

For the calibration, the signals of GNSS, IMU and compass sensors are utilized and the reference measurements will be denoted as ${\tilde{y}}_{k}={\tilde{x}}_{k}$. Thus, the calibration task is the following inference problem

(14) $$\left\{{\tilde{y}}_{k},u_{k}\right\}\to \hat{\theta }=\left[c_{e,RL},c_{d},t_{R},D\right]\;\mathrm{where}\;y_{k}=x_{k}=f\left(x_{k-1},u_{k-1},\theta \right),\;k=k_{0}...K.$$

### 2.3. Demonstration of the Necessity of Model Calibration

**Figure 2:**
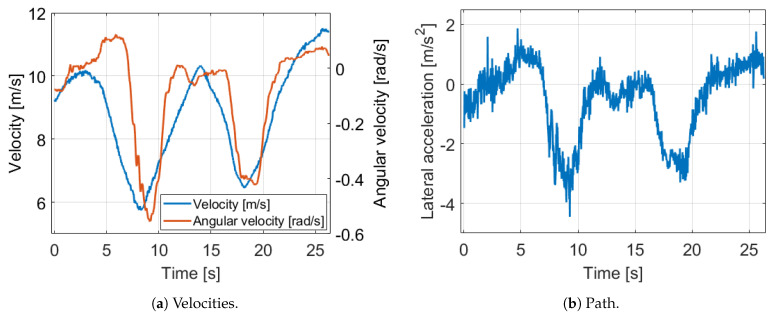
Velocity and acceleration signals of the motivation example.

**Figure 3:**
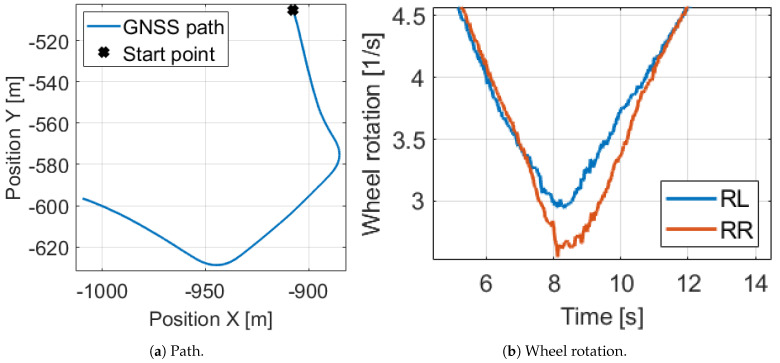
Path and wheel rotation signals of the motivation example.

The effect of the uncalibrated odometry model can be illustrated easily on a 230 m long measurement segment with our Nissan Leaf test vehicle in suburb driving. The signals of the GNSS and IMU sensors are fused to reach reference position and orientation values. The signals of the example can be found in Figure 2 and Figure 3.

The presented odometry model is initialized at the start point with the measurements, and integrated using the wheel rotation measurements. The sideslip and dynamic wheel model is not utilized at this time. The two-wheel model is tested with various settings, which can be found in Table 1. The model settings are compared based on the mean position ($Err_{p}$) and orientation ($Err_{\psi }$) errors calculated from the reference ones. In Case 1, the nominal values are used that available in the vehicle data sheet. The nominal circumferences ($c_{e,i,nom}=2$ m) are the geometry circumference of the wheel without load, and the rear left and right are assumed to be equal. Thus, these values are highly uncertain and result in an enormous 19 m mean position and $12^{\circ }$ mean orientation error.

In Cases 2, 3, 4 and 5 the model is tested with parameters close to the true values one by one. As we can see, every parameter has a high influence and the resulted in error decreases are in the same range. However, Cases 3 and 4 illustrate that the circumference difference is the most significant, despite its extremely low 2 mm value. This is explained by the equation the angular velocity is determined in Equation (5). The difference of the rear wheel velocities are calculated and this value is scaled by the rear track width. Due to the wheel rotations are almost the same in normal driving, (for example Figure 3b shows in the presented measurement segment that in the middle of the bend, when the angular velocity has maximum, the relative difference of the wheel rotations is only 10%), the little difference of the circumferences has high impact. Therefore, we apply an effective and a difference value for wheel parameters instead of separate wheel circumferences. Finally, at the Case 6, when every parameter is set close to its true value, the position is 8× and the orientation error is 6× lower, consequently the odometry model has to be calibrated.

### 2.4. Impact of Dynamic Wheel Circumference and Sideslip

**Figure 4:**
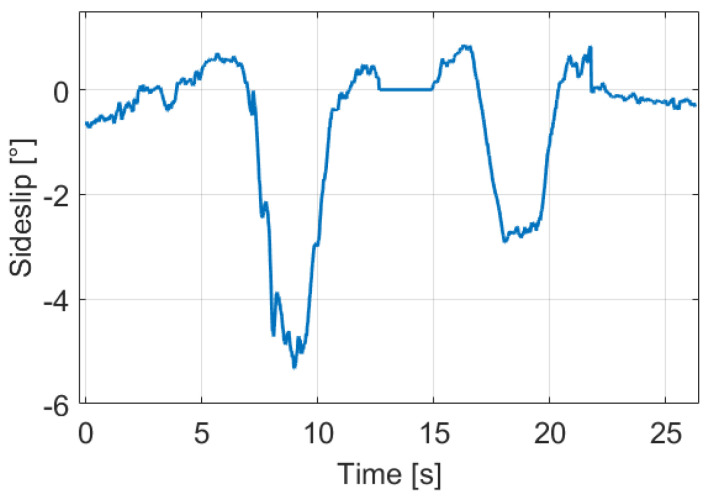
Sideslip signal of the motivation example.

In this section, the impact of the proposed dynamic wheel model and the sideslip to the model calibration is illustrated on the same measurement segment. The estimated sideslip signal can be found in Figure 4, the applied algorithm will be presented in Section 3.

The odometry model is calibrated and tested in the four possible cases (Case 7–10, Table 2) based on the usage of the dynamic wheel model and sideslip values. The calibration method in this example was a genetic algorithm-based optimization, in which the convergence to the optimum can be guaranteed, even in complex non-convex optimization problems, if the computation time is not important. The core problem of the odometry calibration is well illustrated at Case 7, which is similar to the previous one, where the dynamic wheel model and the sideslip is not utilized. However, while in Case 6 the parameters are set close to the true values, in this case, the optimizer provides the setting that results in the lowest error. As we can see, on the presented measurement segment the optimal values differ from the true values of the parameters, but the estimated values are correct because the errors are lower than in Case 6.

The reason can be traced back to several effects. The measurement noises, especially in the initial state of the segment, or the disturbance in the wheel rotation signals, because of the slight wheel slipping, can influence the location of the optimum. These are not modeling problems but filtering issues. However, the deterministic unmodeled effects, such as the sideslip and the dynamic change of the wheel circumference in bends, can result in infeasible parameter identification as well. Furthermore, in Cases 8 and 9 one of the mentioned dynamic effects is taken into account, the calibrated model results in better localization, but the estimated parameters still differ from the true values. However, when every dynamic assumption is included into the odometry model in Case 10, the estimated optimal parameters are close to its true values. It can be validated also with the fact that the position and orientation error is the lowest with the parameter setting of the last case.

**Figure 5:**
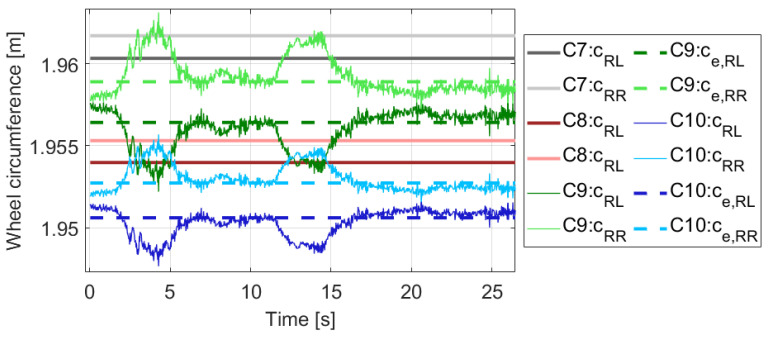
Wheel circumferences of the motivation example.

In summary, the deterministic effects, such as sideslip and dynamic wheel circumference change, are essential for the proper calibration of the odometry models. Neglecting these not only increases the fitting error, but also causes biased parameter estimation regardless of correct convergence. Furthermore, even though the value of *D* is low, do not forget that it is multiplied by the lateral acceleration. The effect of the product in the final case, for example at $3\;m/s^{2}$, is 4 mm in circumference difference, which is also low relative to the circumference of the wheel. However, the difference of the effective circumferences also in the millimeter range, and in the previous section we have seen that its impact is significant. The actual wheel circumferences of Cases 7–10 can be found in Figure 5, and it illustrates that the dynamic part can even result in a change in which wheel is larger at the moment.

## 3. Sideslip Estimation for the Odometry Calibration

Motion estimation for ground vehicles generally based on IMU, gyroscope, wheel encoder, and GNSS sensors. The estimation of quantities e.g., longitudinal velocity, yaw rate are well-researched topics. Though, the proper estimation of some important signals, such as the vehicle sideslip angle remains a challenge. The sideslip is the angle between the lateral ($v_{y}$) and longitudinal ($v_{x}$) velocity of the vehicle. The GNSS sensor measures the longitudinal component, and also it can be estimated with the IMU and wheel encoders. However, measuring the lateral component is difficult or highly expensive, but it can be estimated with the following equation

(15) $${\dot{v}}_{y}=a_{y}-v_{x}\cdot \omega_{z},$$

where $a_{y}$ is the lateral acceleration and $\omega_{z}$ is the yaw rate. The sideslip is based on its integrated value, such as

(16) $$\beta =atan\left(v_{y}/v_{x}\right).$$

The problem is that the value of ${\dot{v}}_{y}$ is low, therefore the effect of sensor errors of the IMU and gyroscope is significant. Any bias or colored noise can results in drift in the lateral velocity, and in the sideslip as well. The problem is more crucial, if continuous value of the sideslip signal is required e.g., in the case of odometry model calibration.

Nevertheless, if we can find the places when the sideslip or in parallel the lateral velocity should be zero, the integration will not diverge. Our proposed method assumes that the effect of sideslip is significant in the odometry process only when the vehicle is cornering, thus the task is to determine the start and endpoint of the bends. With these locations, the estimation can be reset to avoid the drift. For the calculation of these possible zero-crossing places, the curvature of the vehicle path is used up.

### 3.1. Zero-Crossing Based on the Path Curvature

Finding the start and endpoint of the bends is similar to the determination when the vehicle is moving straight. The easiest way to find the straights is to determine a curvature limit, below which the given section of the path is assumed to be straight. The curvature can be calculated with Equation (17), where the curve is parametrized with the $p_{x,k}$ and $p_{y,k}$ position values and assumed to be twice differentiable [49].

(17) $$\kappa_{k}=\frac{{\dot{p}}_{x,k}\;{\ddot{p}}_{y,k}-{\ddot{p}}_{x,k}\;{\dot{p}}_{y,k}}{{\left({{\dot{p}}_{x,k}}^{2}+{{\dot{p}}_{y,k}}^{2}\right)}^{3/2}},$$

(18) $${\dot{p}}_{i,k}=\frac{-0.5p_{i,k-\Delta k}+0.5p_{i,k+\Delta k}}{\Delta t}\;{\ddot{p}}_{i,k}=\frac{0.25p_{i,k-2\Delta k}-0.5p_{i,k}+0.25p_{i,k+2\Delta k}}{\Delta t}$$

**Figure 6:**
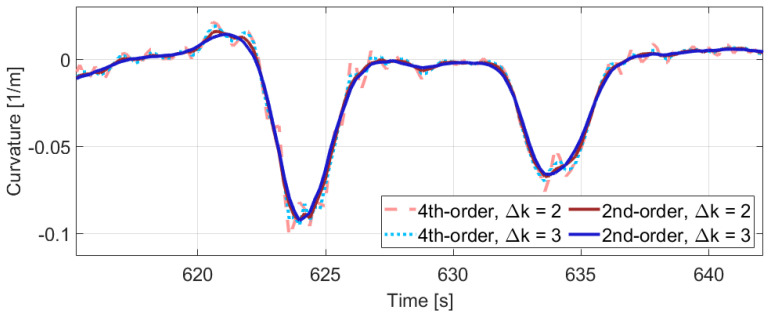
Part of the estimated curvature signal.

The derivatives are calculated with the central finite difference method (18). Since the numerical derivation highlights the impact of noise, not the consecutive GNSS position values are applied in the calculation to smooth the curvature signal. Furthermore, we examined higher-order differentiation, but Figure 6 illustrates that the 2nd-order method with Δk step size of 3 results in the proper estimation. Finally, a velocity limit is also applied, because when the velocity is lower than 1 m/s, the curvature estimation becomes unreliable due to the noisy position measurements.

**Figure 7:**
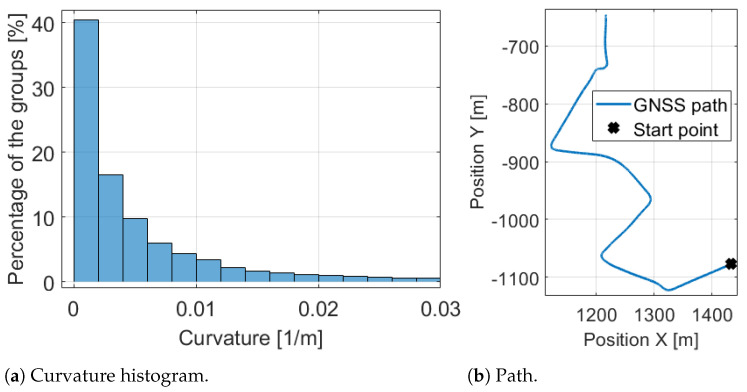
Path and its curvature histogram.

**Figure 8:**
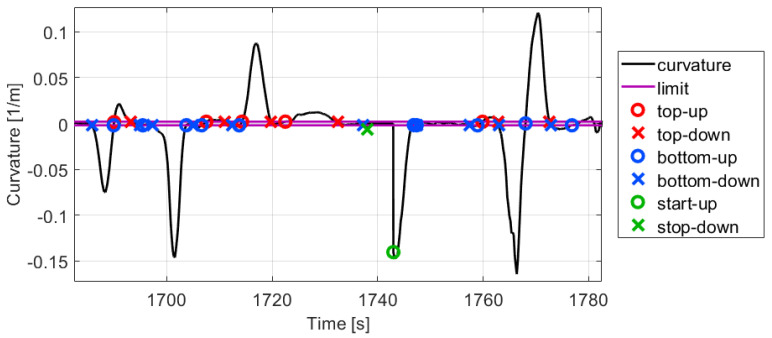
Curvature signal with limit crossings.

For the determination of the mentioned limit for straight the histogram of the absolute curvature values is formulated which can be found in Figure 7a. It shows that 40% of the path points are between 0 and 0.002, therefore the limit is determined as 0.002. It is very informative and means that when the vehicle is moving on a circular path with a higher than 500 m radius is assumed to be straight moving. Figure 8 shows a part of the measurement and illustrates that with the estimated curvature and determined limits the bends clearly appear. The path of the part is also presented in Figure 7b for comparison.

Next, the crossings of the curvature signal at the top (0.002) and bottom (−0.002) limits, and also when the vehicle starts or stops are determined. Using up these crossings the lateral velocity integration is executed between top-up and top-down, bottom-down and bottom-up, and also between the corresponding start and stop crossings.

### 3.2. The Estimated Sideslip

**Figure 9:**
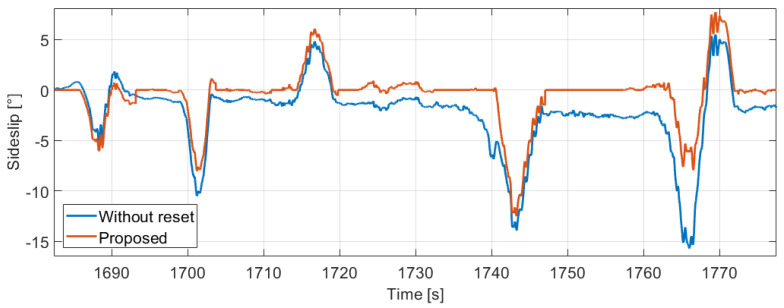
Estimated sideslip signals.

The lateral velocity estimation is performed between the crossings presented before. The advantage of the method is that the velocity integration restarts every time when the curvature signal crosses out the straight limits, thus the effect of previous biases in the derivative of the lateral velocity signal is eliminated. The estimated sideslip of the same part as the previous figures can be found in Figure 9. The signal without any reset in the lateral velocity calculation is also presented (the integration starts at the beginning of the measurement part).

**Figure 10:**
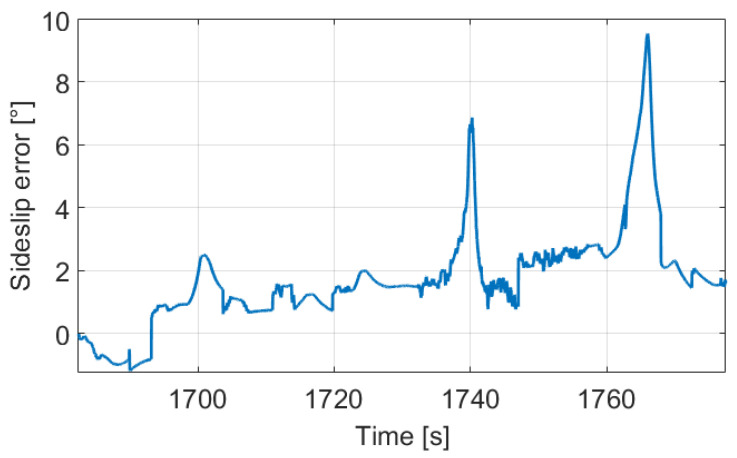
Deviation of the methods.

The mean difference between the signals is only $1.90^{\circ }$, however it corresponds to 50–60% in relative terms when the sideslip is significant (proposed signal is higher than $1^{\circ }$). Moreover, the deviation can be huge in sharp bends as we can see in Figure 10, for example between 1760–1770 s.

**Figure 11:**
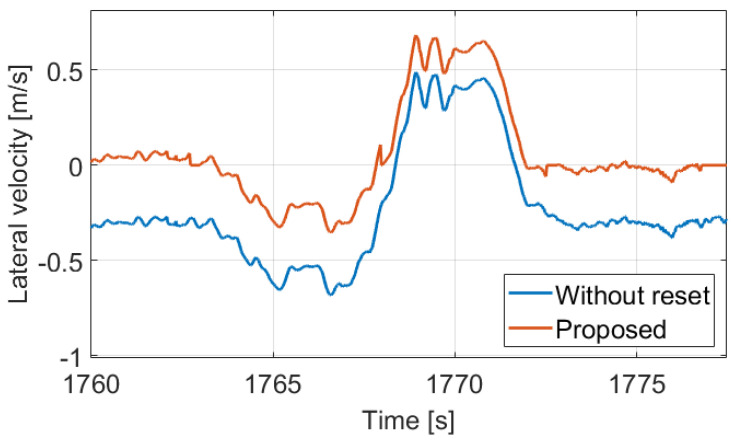
Lateral velocity signals.

The reason is in the calculation of the sideslip, because the same error in the lateral velocity may result in a huge deviation in the sideslip based on the current value of the longitudinal velocity component. Figure 11 shows the lateral velocities between 1762–1772 s, in which the proposed method detects two bends based on the limit crossings. Therefore, the lateral velocity (and in parallel the sideslip) is estimated throughout, and its consequence is that the difference between the velocity signals is constant. The difference value is 0.34 m/s, and it results in $2.42^{\circ }$ sideslip error at the beginning, while $9.54^{\circ }$ at the center of the bend, and also about $2.12^{\circ }$ at the end.

These values show that the proposed resetting method in the lateral velocity estimation has much higher significance than a shift of the lateral velocity with its actual and relative low bias along the vertical axis. Furthermore, the presented algorithm may be further developed due to the faulty limit crossings in the curvature signal, when the vehicle is traveling roughly straight, see for example between 1704–1714 s in Figure 8. However, the estimated sideslip shows that the signal is not diverging in these short time intervals. Moreover, it would be difficult to separate the fail peaks due to the noisy position measurements for example, and the possible deterministic cases for example a lane change or similar movements when a short increase appears in the curvature signal. Nevertheless, these short effects do not significantly affect the calibration process of the odometry model or the localization with it.

## 4. Formulation of the Calibration Algorithm

In Section 2.3, the high impact of the model calibration has been presented. Several possible ideas have been presented in the introduction. The key factor is the assumptions that determine the circumstances of the calibration. For example, if a method operates with pre-programmed paths, the excitation of the system can be determined manually. Information on the excitation can be included in the parameter estimation method and consequently, the unobservability problem may not appear. Furthermore, the applied sensors also influence the methods, for example using expensive sensors such as DGPS or LiDAR the effect of measurement noise is much less significant.

In the development of the calibration algorithm, the universal requirements of the automotive industry are taken into consideration, with which our proposed method handles the most general case. Only cost-effective automotive-grade type sensors are utilized for the parameter estimation, such as GNSS and IMU. Since the algorithm is automatic, it is unnecessary for the vehicle to follow pre-programmed paths, but signals measured during the general motion of the vehicle are used for calibration. Finally, self-calibration may be expected from an autonomous vehicle that operates lifelong, therefore our method does not require any human interventions.

It was mentioned in the introduction that the odometry model calibration can be performed in two different ways. One is when the estimated parameters are handled as a state variable, and the other is to form a regression problem. The proposed method is one of the second type because, due to the cost-effective sensors, the noise of the measurements is significant, which can be mitigated only by optimization over a longer measurement period. The disadvantage of the regression-based calibration is that the odometry model is nonlinear which results in a non-convex optimization. The calibration of the wheel odometry model as a nonlinear least square regression problem is the following,

(19) $${\hat{\theta }}_{opt}=\underset{\theta }{arg\;min}\;\frac{1}{N}\sum_{k=k_{0}}^{k_{0}+N-1}{\left({\tilde{y}}_{k}-y_{k}\left(\theta \right)\right)}^{2},$$

where ${\tilde{y}}_{k}$ contains the measurements, and the $y_{k}\left(\theta \right)$ predictor is the output of the odometry model (8). Since the objective function can not be formed as a linear function of parameter vector θ, the basic least square method cannot be applied. This type of optimization problem is difficult to solve in general [50].

This can be well illustrated also by the approaches in the field of odometry calibration, since all of the methods apply some simplifications to handle the difficulties of solving the general nonlinear regression problem. A separation into two linear in [32], and into a linear and nonlinear regression in [31] are presented. In these cases, the orientation equation is optimized first, and its result is utilized in the position equations. This technique was implemented also, but the parameter estimation was highly biased. The problem is that in the first step only the orientation measurements are applied, whose noise is significant because the correct determination of the absolute orientation is difficult outdoor. This results in bias which is propagated to the estimation of the remaining components inducing an unfeasible identification task. Therefore, these methods require excellent orientation estimations, which is complicated to guarantee with cost-effective sensors outdoors. The method is improved with an iterative loop in [33] to increase the convergence to the true parameter values, however it is an indoor application for mobile robots. Another simplification of the regression problem can be found in [18], where the nonlinear estimation problem is handled with linearized system dynamics and integrated prediction error minimization. The disadvantage of the linearization is that the calculated Jacobian with respect to the parameters is only valid close to the actual predicted trajectory. However, this may be highly inaccurate especially at the beginning of the optimization due to the imprecise initial guess of the parameter values.

### 4.1. Gauss–Newton Based Nonlinear Least Square Method for Calibration

To solve the nonlinear least square (LS) problem, numerical search is required. The two universal approaches are the Newton–Raphson and Gauss–Newton (GN) methods. Both techniques handle the nonlinearity with the first-order Taylor approximation. In the first, the optimization criterion is specifically to set the derivative of the $V_{N}$ objective function to zero, thus the Taylor’s series of this is formed. The parameters are concentrated in the angular velocity Equation (5) in a special situation such that the sum of the resulting components divided by the $t_{R}$ track width is the yaw rate. The consequence of this is that several local optimums exist in the parameter field of the optimization task (19). The assumption is examined in detail in our previous paper [51], where the interaction of the track width and wheel circumferences are illustrated. Because of this non-convex case, the Newton–Raphson technique is not preferred.

In contrast to Newton–Raphson, the GN method approximates the $y_{k}\left(\theta \right)$ predictor in the following way:

(20) $$y_{k}\left(\theta \right)\approx y_{k}\left(\theta_{i-1}\right)+{\left.\frac{\partial y_{k}\left(\theta \right)}{\partial \theta }\right|}_{\theta_{i-1}}\left(\theta -\theta_{i-1}\right).$$

Introducing the following new variables

(21) $${\tilde{z}}_{k}\left(\theta_{i-1}\right):={\tilde{y}}_{k}-y_{k}\left(\theta_{i-1}\right),\;\vartheta :=\theta -\theta_{i-1},\;\phi_{k}\left(\theta_{i-1}\right):={\left.\frac{\partial y_{k}\left(\theta \right)}{\partial \theta }\right|}_{\theta_{i-1}},$$

a locally linearized LS problem can be formed such as,

(22) $$V_{N}\left(\vartheta \right)=\frac{1}{N}\sum_{k=k_{0}}^{k_{0}+N-1}{\left({\tilde{z}}_{k}\left(\theta_{i-1}\right)-z_{k}\left(\theta_{i-1}\right)\right)}^{2}=\frac{1}{N}\sum_{k=k_{0}}^{k_{0}+N-1}{\left({\tilde{z}}_{k}\left(\theta_{i-1}\right)-\vartheta^{T}\phi_{k}\left(\theta_{i-1}\right)\right)}^{2}.$$

In the locally linear LS problem, the ${\tilde{z}}_{k}\left(\theta_{i-1}\right)$ “measurement” applied for the parameter estimation is the difference of the original physical measurement (${\tilde{y}}_{k}$) and the output of the model with the actual parameters ($y_{k}\left(\theta_{i-1}\right)$). The regressor $\phi_{k}\left(\theta_{i-1}\right)$ contains the Taylor-approximation of the predictor, and the estimated ϑ parameter is the variation of the original θ parameter vector. The local behavior illustrates that every component is a function of $\left(\theta_{i-1}\right)$, which is the previous value of the original parameter vector. This also means that the parameter estimation can be performed only in an iterative loop (*i* is the iteration number), and $\left(\theta_{0}\right)$ initial guess for the parameters is necessary. The advantage of this method is that the optimal estimated ϑ parameter is resulted in by the basic LS solution, such as

(23) $${\hat{\vartheta }}_{opt}=\underset{\vartheta }{arg\;min}\;V_{N}\left(\vartheta \right)={\left(\Phi {\left(\theta_{i-1}\right)}^{T}\Phi \left(\theta_{i-1}\right)\right)}^{-1}\Phi {\left(\theta_{i-1}\right)}^{T}\tilde{Z}\left(\theta_{i-1}\right),$$

where Φ and $\tilde{Z}$ matrices constructed from $\phi_{k}\left(\theta_{i-1}\right)$ and ${\tilde{z}}_{k}\left(\theta_{i-1}\right)$ respectively. Rewriting the equation with the original quantities and the ${\hat{\vartheta }}_{opt}={\hat{\theta }}_{i}-{\hat{\theta }}_{i-1}$ LS solution, the iterative parameter estimation with GN method can be expressed, such as

(24) $${\hat{\theta }}_{i}={\hat{\theta }}_{i-1}+{\left(\Phi {\left(\theta_{i-1}\right)}^{T}\Phi \left(\theta_{i-1}\right)\right)}^{-1}\Phi {\left(\theta_{i-1}\right)}^{T}\left(\tilde{Y}-Y\left(\theta_{i-1}\right)\right),$$

where $\tilde{Y}$ and $Y(\theta_{i-1})$ matrices constructed from ${\tilde{y}}_{k}$ and $y_{k}\left(\theta_{i-1}\right)$ respectively, and the algorithm starts with ${\hat{\theta }}_{0}$ initial parameter given by the user.

### 4.2. Odometry Calibration with Gauss–Newton Method and Integrated Kalman-Filtering

The odometry model calibration with the general GN method suffers from three main problems. The first is any unmodeled effect can result in biased parameter estimation due to the calibration is formed as a numerical minimization problem. The second is that the optimization problem is non-convex with several local minima, thus the convergence to the global optimum is always questionable and also depends on the initial guess of the parameter vector. Finally the third connects to the uncertainty of the measurements utilized for the parameter estimation. It is contributed by the probability learning theory that noisy measurements result in noisy parameter estimation. However, because the ${\hat{y}}_{k}\left(\theta \right)$ predictor in this regression problem is a dynamic system model (the odometry model) the measurement uncertainty has a high impact. At the beginning of the section, some possible handlings of the problems are mentioned in the presented works [18,31,32,33] in the field of mobile robots. Due to the improper orientation measurements in the outdoors and the complexity of our odometry model other improvement is necessary.

One of the advantages of the physical modeling of the wheel odometry is initial parameter values can be determined easily. For initial wheel circumference and track width, the datasheet values can be utilized and for circumference difference and load transfer coefficient, zero may be correct. From the modeling perspective, the impact of the lateral dynamics has been presented and consequently included in the model. The only remaining effects can be the wheel slips and the longitudinal dynamics. However, these have low significance to the odometry model under normal driving and weather conditions, only resulting in a slight bias that can appear in the calibration. The inaccurate pose measurements used for the estimation are the core problem, which needs to be handled. The effect of the uncertainty of the pose measurements can be mitigated by optimizing over a longer horizon. The main problem is the actual inaccuracy at the start point of the regression-based optimization. In general regression problems, when the estimated function is static, this type of problem does not occur. However, forming the objective function of a dynamic system (22), the $y_{k}\left(\theta_{i-1}\right)$ predictor needs an initial state at the $k_{0}$ start point. This component appears in the last $\left(\tilde{Y}-Y\left(\theta_{i-1}\right)\right)$ part of the estimation Equation (24).

Substituting the wheel odometry model into the optimization equations, the problem can be illustrated clearly. Due to the fact that every state variable is measured directly with GNSS and compass sensors, the output is equal with the states as $y_{k}=x_{k}$. Thus, the matrix in the mentioned last part is structured as follows,

(25) $$\tilde{Z}=\tilde{Y}-Y\left(\theta_{i-1}\right)=\left[\begin{array}{c} {\tilde{x}}_{k0}-x_{k0}\left(\theta_{i-1}\right)\\ {\tilde{x}}_{k0+1}-x_{k0+1}\left(\theta_{i-1}\right)\\ \vdots \end{array}\right]=\left[\begin{array}{c} {\tilde{x}}_{k0}-f\left(x_{k0-1},u_{k0-1},\theta_{i-1}\right)\\ {\tilde{x}}_{k0+1}-f\left(x_{k0},u_{k0},\theta_{i-1}\right)\\ \vdots \end{array}\right]$$

where $f(x_{k-1},u_{k-1},\theta_{i})$ is the wheel odometry model (8). For the construction of the minimization problem the only way is to initialize the odometry model in the first component of the matrix with the previous measurement value, such as

(26) $$f\left(x_{k0-1},u_{k0-1},\theta_{i-1}\right)=f\left({\tilde{x}}_{k0-1},u_{k0-1},\theta_{i-1}\right).$$

However, if the measurement ${\tilde{x}}_{k0-1}={\left[p_{x,k0-1},p_{y,k0-1},\psi_{x,k0-1}\right]}^{T}$ is incorrect, even the perfectly calibrated model will diverge from the further measurements. Thus, the imprecise pose measurements result in bias parameter estimation. Although the uncertainty of the measurements applied for the parameter estimation is rather a filtering problem, it also has to be handled in the parameter estimation to reach a properly calibrated model.

In summary, the possible wheel slip or longitudinal dynamics, and the uncertain pose measurements(utilized as initial state) corrupt the $x_{k}\left(\theta \right)$ predictor (which is the odometry function) in the parameter estimation. Reducing these negative effects the proposed method modifies the predictor in a unique way. The odometry model (8) can be separated into two parts, such as

(27) $$x_{k}\left(\theta_{i-1}\right)=f\left(x_{k-1},u_{k-1},\theta_{i}\right)=\underset{x_{k-1}}{\underbrace{\left[\begin{array}{c} p_{x,k-1}\\ p_{y,k-1}\\ \psi_{k-1} \end{array}\right]}}+\underset{f_{disp}\left(\psi_{k-1},u_{k-1},\theta_{i-1}\right)}{\underbrace{\left[\begin{array}{c} v_{k-1}\cdot \cos\left(\psi_{k-1}+\omega_{k-1}/2+\beta_{k-1}\right)\\ v_{k-1}\cdot \sin\left(\psi_{k-1}+\omega_{k-1}/2+\beta_{k-1}\right)\\ \omega_{k-1} \end{array}\right]}},$$

where the first is the previous pose, and $f_{disp}\left(\psi_{k-1},u_{k-1},\theta_{i-1}\right)$ is the displacement between the time steps. Since the initial state noise and the possible unmodeled dynamics corrupt the predictor, the $x_{k-1}$ component is replaced with ${\hat{x}}_{k-1}$ which is a filtered value. Thus, the avoidance of the divergence in the predictor results in biased parameter estimation.

Consequently, the proposed unique addition to the GN parameter estimation algorithm is an inner Kalman-filtering whose filtered states are included in the generation of the LS problem. Due to the nonlinear model, the method of Extended Kalman-filter is applied. This type of filter has two steps, the first is the prediction based on the odometry model with the previous parameters, and wheel rotations as inputs, such as

(28) $${\hat{x}}_{k}^{-}=f\left({\hat{x}}_{k-1},u_{k-1},\theta_{k-1}\right),\;\;\Sigma_{k}^{-}=F_{k}\Sigma_{k-1}F_{k}^{T}+P,$$

where $\Sigma_{k}$ is the covariance of the states and it is also propagated. The covariance calculation requires the Jacobian of the applied function which is computed using the previous filtered states as

(29) $$F_{k}={\left.\frac{\partial f(x_{k},u_{k},\theta_{k-1})}{\partial x}\right|}_{x_{k}={\hat{x}}_{k-1},u_{k}={\hat{u}}_{k-1}}.$$

The filter is controlled by the $G_{k}$ Kalman-gain which ensures the optimal estimation and minimal covariance. The calculation is the following

(30) $$G_{k}=\Sigma_{k}^{-}{\left(\Sigma_{k}^{-}+M\right)}^{-1},$$

in which the *P* process and *M* measurement covariance matrices which are tuning variables. The second step is the innovation, in which the pose measurements are utilized to improve the prediction, such as

(31) $${\hat{x}}_{k}={\hat{x}}_{k}^{-}+G_{k}\left({\tilde{x}}_{k}-{\hat{x}}_{k}^{-}\right)\;\;\Sigma_{k}=\left(I-G_{k}\right)\Sigma_{k}^{-}.$$

This filtered state is included into the predictor (27), such as

(32) $$x_{k}\left(\theta_{i-1}\right)=x_{k-1}+f_{disp}\left(\psi_{k-1},u_{k-1},\theta_{i-1}\right)={\hat{x}}_{k-1}+f_{disp}\left({\hat{\psi }}_{k-1},u_{k-1},\theta_{i-1}\right),$$

which is used to construct the local LS problem in the following way,

(33) $$\tilde{Z}=\tilde{Y}-Y\left(\theta_{i-1}\right)=\left[\begin{array}{c} {\tilde{x}}_{k0}-x_{k0}\left(\theta_{i-1}\right)\\ {\tilde{x}}_{k0+1}-x_{k0+1}\left(\theta_{i-1}\right)\\ {\tilde{x}}_{k0+2}-x_{k0+2}\left(\theta_{i-1}\right)\\ \vdots \end{array}\right]={\left[\begin{array}{c} {\tilde{x}}_{k0}-{\tilde{x}}_{k0-1}+f_{disp}\left({\tilde{\psi }}_{k0-1},u_{k0-1},\theta_{i-1}\right)\\ {\tilde{x}}_{k0+1}-{\hat{x}}_{k0}+f_{disp}\left({\hat{\psi }}_{k0},u_{k0},\theta_{i-1}\right)\\ {\tilde{x}}_{k0+2}-{\hat{x}}_{k0+1}+f_{disp}\left({\hat{\psi }}_{k0+1},u_{k0+1},\theta_{i-1}\right)\\ \vdots \end{array}\right]}_{3N\times 1}.$$

The advantage of the Kalman-filter (KF) addition is that the corruption of the $x_{k}\left(\theta \right)$ predictor by the mentioned uncertain effects can be reduced which improves the accuracy of the parameter estimation. In a theoretical manner, this addition can be interpreted as a trade-off between the well-known prediction (when the output is based only on the previous inputs) and simulation (when the previous outputs also applied for the actual output calculation) usage of the predictor model, which considerations have a central role in the identification theory [52]. The last addition to the method is a *W* weight matrix which is used to compensate for the different magnitudes of the position and orientation equation noise. The equation of the GN-KF iterative parameter estimation is the following:

(34) $${\hat{\theta }}_{i}={\hat{\theta }}_{i-1}+{\left(\Phi {\left(\theta_{i-1}\right)}^{T}W\Phi \left(\theta_{i-1}\right)\right)}^{-1}\Phi {\left(\theta_{i-1}\right)}^{T}W\left(\tilde{Y}-Y\left(\theta_{i-1}\right)\right).$$

**Figure 12:**
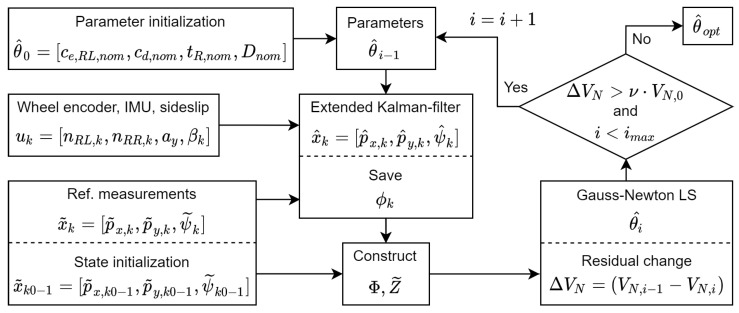
Process of the Gauss–Newton (GN)-Kalman-filter (KF) iterative parameter estimation method.

The whole process is illustrated in Figure 12, and the method will be indicated by GN-KF in the following.

The method is an iterative algorithm, thus a stopping condition needs to be defined. A possible choice can be to monitor the $\Delta V_{N}$ change of the sum of residuals and determine a lower limit below which the estimation is stopped. In our method, the limit is computed using the $V_{N,0}$ initial sum of residuals, and a ν rate parameter. Furthermore, the maximum number of iterations also limited. If sufficient computing capacity is available, the ν parameter may be set to zero.

### 4.3. The Calibration Architecture

It has also been mentioned that our proposed calibration method is automatic, and operates with signals measured during normal traveling of the vehicle on any path. For the most general behavior, no constraints are applied, such as to estimate the parameters only between two standstill or given path segments. However, in the forming of the objective function for the LS problem (22), the number of measurement points is *N* from a $k_{0}$ given start index. Thus, a horizon length in which the model is being calibrated should be determined.

The calculation of the optimal *N* is based on two opposing effects that relate to the uncertainty of the measurements used for the estimation. In general, it is evident that the impact of measurement noise is decreasing with the involving multiple data points into an LS regression problem. In our case, this may not be able to succeed due to the dynamic behavior of the predictor. In the previous section, the impact of the imprecise initial state is examined in detail which is why the Kalman-filtering has been integrated into the estimation loop. Although this handles the problem by reducing the divergence, the uncertainty is not eliminated completely, and its negative impact is increasing if the estimation horizon is longer. Therefore, the proper value of *N* is determined with empirical tests, the resulted in value is N=1350 which corresponds to a path length of 300 m and 33.75 s in time. The calibration architecture is a moving window procedure, where the parameter estimation is performed in every 10 s on the actual subtrace. The estimated model parameters are stored and after a given number of estimations, the average is calculated which results in the calibrated model.

### 4.4. Tuning of the Weight in the Gauss–Newton Method

The well-known LS problem formulation is only a special case of the Generalized LS technique, in which the assumption on the error term has the same variance in each observation is taken. The presented parameter estimation Formula (34) is another special case of the Generalized LS method because the variance of the observed values are assumed to be unequal, but without correlations among them. This means that the off-diagonal elements of *W* are zero. Furthermore, the $\sigma_{x_{k}}$ variance vectors of the ${\tilde{x}}_{k}$ measurements, which resulting from measurement inaccuracies of GNSS and compass sensors, are assumed be be constant, $\sigma_{{\tilde{x}}_{k}}=\sigma_{\tilde{x}}$. Therefore, only the components of the state variance vector $\sigma_{\tilde{x}}=\left[\sigma_{{\tilde{p}}_{x}},\sigma_{{\tilde{p}}_{y}},\sigma_{\tilde{\psi }}\right]$ should be determined. Thus, the *W* weight matrix is formulated, such as

(35) $$W={\left[\begin{array}{ccccccc} 1/\sigma_{{\tilde{p}}_{x}} & 0 & 0 & 0 & 0 & 0 & \cdots \\ 0 & 1/\sigma_{{\tilde{p}}_{y}} & 0 & 0 & 0 & 0 & \cdots \\ 0 & 0 & 1/\sigma_{\tilde{\psi }} & 0 & 0 & 0 & \cdots \\ 0 & 0 & 0 & 1/\sigma_{{\tilde{p}}_{x}} & 0 & 0 & \cdots \\ 0 & 0 & 0 & 0 & 1/\sigma_{{\tilde{p}}_{y}} & 0 & \cdots \\ 0 & 0 & 0 & 0 & 0 & 1/\sigma_{\tilde{\psi }} & \cdots \\ \vdots & \vdots & \vdots & \vdots & \vdots & \vdots & \ddots \end{array}\right]}_{3N\times 3N}.$$

However, in the regression problem the reciprocal of the vector components are incorporated. Furthermore, only the ration among them matters in the calibration process. In summary, in the GN based regression $\sigma_{{\tilde{p}}_{x}}=\sigma_{{\tilde{p}}_{y}}=1$ are applied, and the only remaining tuning parameter is the $\sigma_{\tilde{\psi }}$. Because of the easier illustration, $w_{\tilde{\psi }}=1/\sigma_{\tilde{\psi }}$ is tuned and named as the weight of the orientation equation.

In our calibration example, the *W* weight matrix has two objectives. The first is evident to guarantee the convergence to the optimum. The second, which is more complex, is to improve the generalization capability of the method. This attribute arises due to the calibration is performed separately on subtraces. If $w_{\tilde{\psi }}$ is set too low, the orientation equation will not have any importance in the model calibration. This is the consequence of the numerical optimization because the LS method minimizes the $V_{N}$ sum of residuals (22), and the orientation is measured in rad, while the positions are in *m* which numerical values are significantly higher in case of car measurements. Therefore, low $w_{\tilde{\psi }}$ results in a locally optimal calibration which minimizes mainly the position error on the actual subtrace. However, the resulted in setting may perform inaccurately on other subtraces, which reflects on the poor generalization capability.

**Figure 13:**
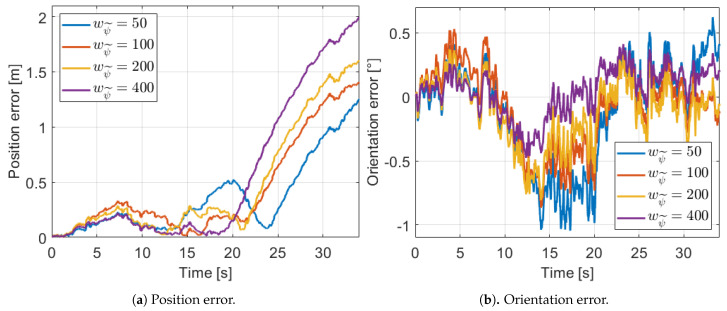
Errors with various $w_{\tilde{\psi }}$ settings in the estimation horizon.

**Figure 14:**
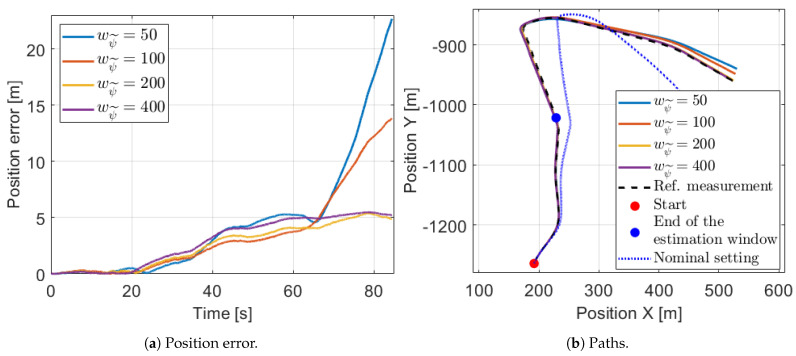
Position signals with various $w_{\tilde{\psi }}$ settings in a longer horizon.

This effect is illustrated in the following example, in which the model calibration on a given subtraces is presented. Four different $w_{\tilde{\psi }}$ values are examined from 50 to 400. The position and orientation errors with the calibrated models can be found in Figure 13 and the estimated parameters and mean error values in Table 3. The method can converge from the uncertain initial setting (shown in Figure 14b as nominal), because with the estimated parameters the errors are quite low. Estimation with $w_{\tilde{\psi }}<50$ is also tested, but the errors were significantly higher. Therefore, the calibration can be guaranteed with $w_{\tilde{\psi }}>50$ setting.

In the last two columns of the table, the effect of the $w_{\tilde{\psi }}$ weight is shown. As it is expected, with the increasing of the weight the orientation error decreases, but in parallel the position error increases. Thus, it is difficult to determine the optimal value of $w_{\tilde{\psi }}$. However, it has been also mentioned that this tuning parameter also responsible for the generalization capability. It is critical because some of the estimated parameters with various $w_{\tilde{\psi }}$ settings significantly differ, although the errors with the various calibrated models are in the same relatively low range. The most decided difference can be observed in the change of the $t_{R}$ and *D* parameter. The track width increases significantly and in parallel, the load transfer coefficient decreases while the orientation error decreases only slightly. It means that the two parameters compensate each other in a unique way, however, this effect highly depends on the actual measurement segment.

The assumption can be validated if the calibrated model is tested on further measurements. For the best illustration the same measurement, on which the calibration is performed, is used but the test is executed on a longer horizon. The position errors in an 85 s long segment are shown in Figure 14a, which illustrates well the effect of the generalization capability. The models perform in a similar manner until 60 s when a sharp bend follows as it can be seen in Figure 14b. After that point, the four calibrated models with various $w_{\tilde{\psi }}$ values differ significantly from each other. Only the models with higher orientation weight can not diverge from the reference measurements, thus these have appropriate generalization capability.

However, this is one example and the optimal value for $w_{\tilde{\psi }}$ can be determined only by several empirical tests. The whole calibration algorithm was tested with $w_{\tilde{\psi }}$ between 100–400, and the optimal setting was achieved by 200. Thus, the components in the *W* matrix are

(36) $$\frac{1}{\sigma_{{\tilde{p}}_{x}}}=\frac{1}{\sigma_{{\tilde{p}}_{y}}}=1\;\;\frac{1}{\sigma_{\tilde{\psi }}}=w_{\tilde{\psi }}=200.$$

### 4.5. Tuning of the Covariances in the Kalman-Filtering

In general, the covariances of the Kalman-filter should be adjusted to the sensor measurement uncertainty. However, the main reason for the Kalman-filter integration to the GN parameter estimation loop is to compensate for the divergence resulting from the uncertainty of the state (${\tilde{x}}_{k0-1}$) with which the calibration problem is initialized at the beginning of the estimation window. It has been mentioned also that the integration of the KF is realizable as a manageable trade-off between the use of the raw prediction and the simulation structure in the $x_{k}\left(\theta \right)$ predictor, such as

(37) $$\mathrm{simulation}\;\mathrm{structure}:x_{k}\left(\theta_{i-1}\right)=x_{k-1}+f_{disp}\left(\psi_{k-1},u_{k-1},\theta_{i-1}\right),$$

(38) $$\mathrm{prediction}\;\mathrm{structure}:x_{k}\left(\theta_{i-1}\right)={\tilde{x}}_{k-1}+f_{disp}\left(\psi_{k-1},u_{k-1},\theta_{i-1}\right),$$

and our proposed structure, where the fused values from the presented Kalman-filter (31) are utilized

(39) $$\mathrm{proposed}\;\mathrm{structure}:x_{k}\left(\theta_{i-1}\right)={\hat{x}}_{k-1}+f_{disp}\left(\psi_{k-1},u_{k-1},\theta_{i-1}\right).$$

Therefore, in our special case with the tuning of the process *P* and measurement *M* covariance matrices in the KF, the ratio between the two structures can be controlled.

**Figure 15:**
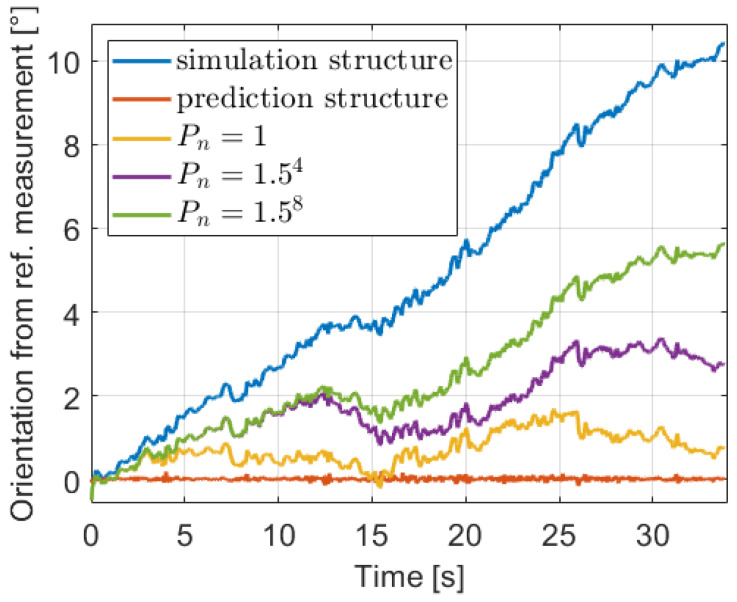
Orientation with various covariances.

The consideration that not the specific values but the ratio of these are important can be applied also in this case. Thus, the *M* matrix is defined as constant and the *P* is such as

$$M=\left[\begin{array}{ccc} 1 & 0 & 0\\ 0 & 1 & 0\\ 0 & 0 & 0.1 \end{array}\right]\;P=\left[\begin{array}{ccc} 0.01 & 0 & 0\\ 0 & 0.01 & 0\\ 0 & 0 & 0.0001 \end{array}\right]/P_{n},$$

because between two time steps the output of the odometry model can change only in the cm range due to the low $T_{s}=0.025$ s sampling time. With this setting, the orientation signals, relative to the reference for better illustration, in the first iteration of the estimation in the previously presented subtrace are presented with various $P_{n}$ value in Figure 15.

The predictor with the mentioned prediction and simulation structures are also shown in the first iteration. With the raw prediction structure, the signal is close to the reference, which means that the $V_{N}$ sum of residuals (22) to be minimized is very low, thus the optimization gets stuck at the beginning of the estimation. While with the simulation structure the signal is relatively high, consequently the norm is high and the convergence is difficult. However, tuning the $P_{n}$ value the $x_{k}\left(\theta \right)$ predictor balances between the two structure approaches. The determination of the optimal $P_{n}$ is based on experimental tests, and a varying method is proposed due to the supposition that the model becomes better and better in the iterations of the GN-KF method. Thus, the value is equal to $P_{n}=1/1.5^{i}$, where *i* is the iteration number in the parameter estimation algorithm (34).

## 5. Measurement Data for the Calibration

### 5.1. Test Vehicle, Measurement and Subtrace Selection

The test vehicle was a Nissan Leaf electric compact car that is equipped with automotive-grade GNSS, compass, and IMU sensors. From the vehicle CAN bus the wheel encoder signals were also saved. The sampling was is 40 Hz.

**Figure 16:**
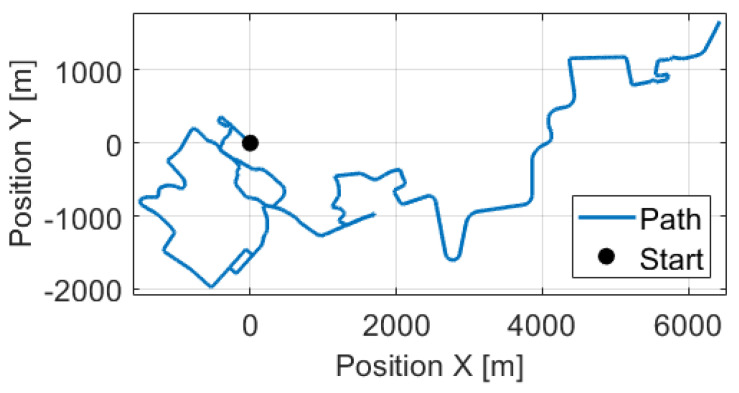
Path of the used measurements.

**Figure 17:**
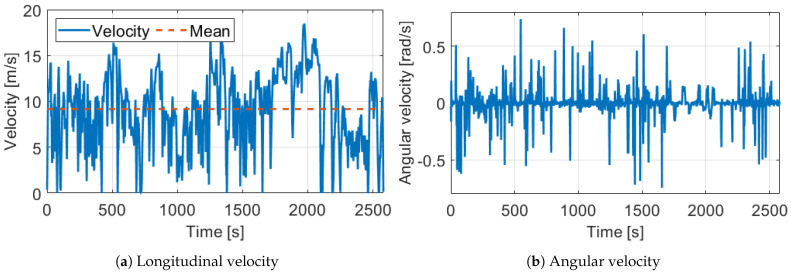
Velocities of the used measurement.

**Figure 18:**
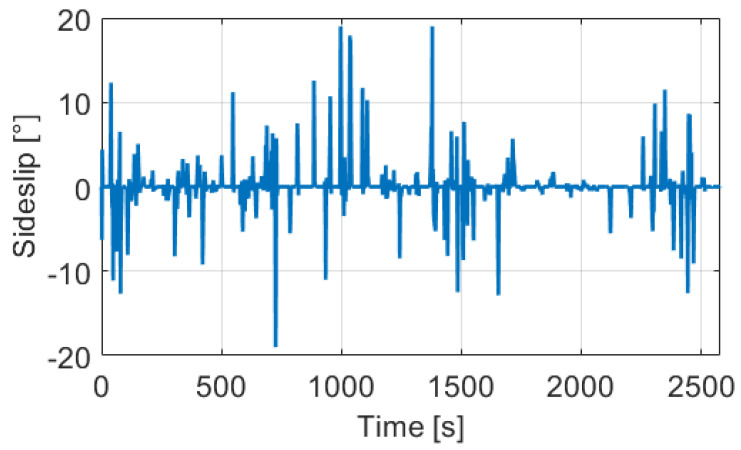
Estimated sideslip of the used measurements.

The test track was a 23.64 km long route in suburb and city driving with full traffic, the path can be found in Figure 16. In the design of the measurement route, the main point of view was the diversity to test the robustness of the proposed odometry model. Therefore, the track contained several sharp, and large curved bends, two roundabouts, and lots of crossroads. Following these elements the velocity signals ( Figure 17) were also varied, the longitudinal maximum was 18.72 m/s, the mean was 9.16 m/s while the angular velocity was between −0.8 rad/s and 0.8 rad/s. The estimated sideslip with the method presented in Section 3 can be found in Figure 18. The signal in the turning cases with low speed could reach higher values, such as 10–20${}^{\circ }$, but when the velocity was significant the sideslip remained lower than $3^{\circ }$.

At the calibration architecture, it has been mentioned that the parameter estimation was executed in a moving window procedure on N=1350 measurement points in each subtrace. The shift between the consecutive windows was 10 s resulting 255 different subtraces. From these, the only ones where the angular velocity was higher than 0.15 rad/s were selected for parameter estimation due to observability issues. Examining the proposed odometry model, it can be seen that the effect of $c_{d}$, $t_{R}$ and *D* parameters mainly appeared in the angular velocity Equation (5). Therefore, the estimation of these parameters was feasible only if the angular velocity was significant. With this constraint, the number of selected subtraces was 183.

Due to the linearization in the GN method, initial guesses for the parameters were necessary. For initial wheel circumference and track width, the datasheet values can be utilized and for circumference difference and load transfer coefficient, zero may be correct. Thus,

(40) $${\hat{\theta }}_{0}={\left[c_{e,RL,nom},c_{d,nom},t_{R,nom},D_{nom}\right]}^{T}={\left[2,0,1.6,0\right]}^{T}$$

### 5.2. Reference Pose Measurement

The measurements used for the estimation were reached from GNSS, compass, and IMU sensor. The pose could be measured directly with the first two, although these signals were assumed to be noisy but unbiased. In contrast, the pose computation from the acceleration and angular velocity measurements with the IMU was generally biased but the noise was lower. Consequently, these measurements were suited for sensor fusion. Thus, before the parameter calibration, the GNSS/compass and IMU signals were fused with a Kalman-filter. This filtering problem is well-explored, our implemented method similar to [53]. The fused pose values were denoted with ${\tilde{x}}_{k}$, and mentioned as measurements for the calibration, or reference measurements.

## 6. Results of the Proposed Method

### 6.1. Illustration of the Iterative GN-KF Estimation Method

**Figure 19:**
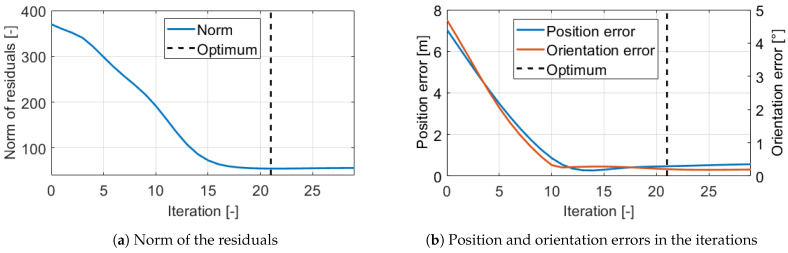
Estimation errors.

The estimation with the iterative GN-KF method is illustrated on the same subtrace which is examined in the tuning sections. In those examinations, the mean position and orientation errors are shown, however the optimization operates with the weighted norm of the objective function (22), also known as the norm of the residuals. The evolution of this can be found in Figure 19a, and also the pose errors in the iterations are shown in Figure 19b. The stopping condition was set to ε=0.003 which resulted in optimal estimation after 21 iterations because in the next the norm increased slightly.

**Figure 20:**
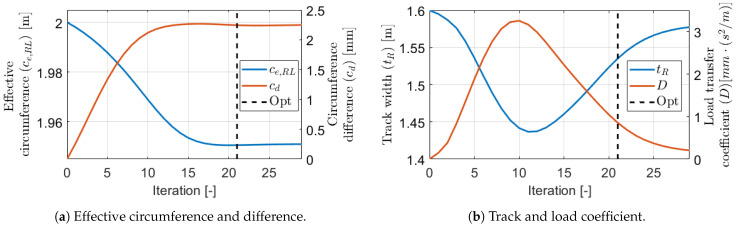
Estimated parameters in the iterations.

The optimization started from the ${\hat{\theta }}_{0}$ initial parameter guess. The parameters in the iterations can be found in Figure 20. The effective circumference and circumference difference smoothly converged to steady-state values. However, the evolution of the track width and the load transfer coefficient was surprising. The signals did not converge in such a way as the other ones but ran smoothly opposite to each other. The same phenomenon has been mentioned already when the tuning of the orientation weight was examined. This evolution of the parameters was not a problem, because the optimization should have reached an optimum because the pose errors were extremely low. The mean position error with the optimal calibration was 0.47 m which corresponded to only 0.18% relative error in this 255 m long subtrace, and 15 times lower than the error with the nominal setting. Probably the strange evolution is due to the unique interaction of the parameters, since all three $c_{d}$, $t_{R}$, and *D* parameters affected the angular velocity.

### 6.2. Parameter Estimation Results

The GN-KF estimation algorithm was executed on every selected subtrace. Although the Kalman-filtering was integrated into the estimation loop to mitigate the divergence of the predictor, in several subtraces the estimated parameters were not valid. For example, the track width at the optimum was more than 2 m or the value of *D* parameter was negative. In these cases, the uncertainty of the measurements used as the initial state at the beginning of the estimation window or the possible wheel slip not only corrupted the parameter estimation but also made the calibration impracticable, regardless of the applied method. This was the trade-off if only cost-effective sensors were applied. However, the calibration parameters had clear physical content, therefore bounds could be determined on which subtrace calibration results to include in the computation of the final stable parameter values. The $t_{R}$ track width was bounded only with 1.1 m lower, and 2.1 m upper limits calculated from the datasheet value and 0.5 m tolerance range. With this restriction, 144 subtrace results remained.

**Figure 21:**
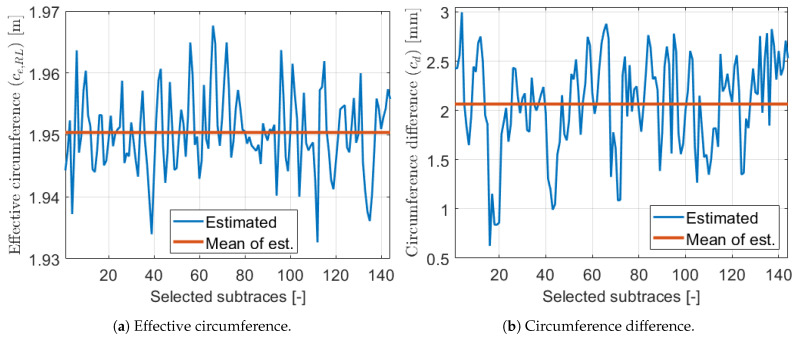
Estimated parameters 1.

The estimated circumference parameter values of the selected subtraces can be found in Figure 21. The effective values were within a 3 cm range while the circumference differences are around 2 mm. Although this low value was only 0.1% of the effective circumference values, the motivation example in Section 2.3 illustrates its high impact in the wheel odometry model, thus this result is important.

**Figure 22:**
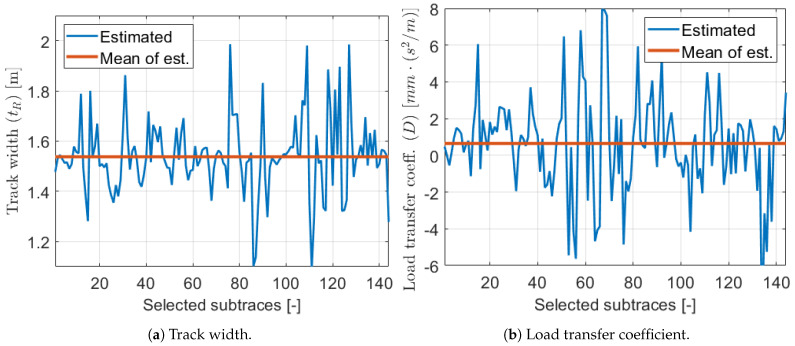
Estimated parameters 2.

**Figure 23:**
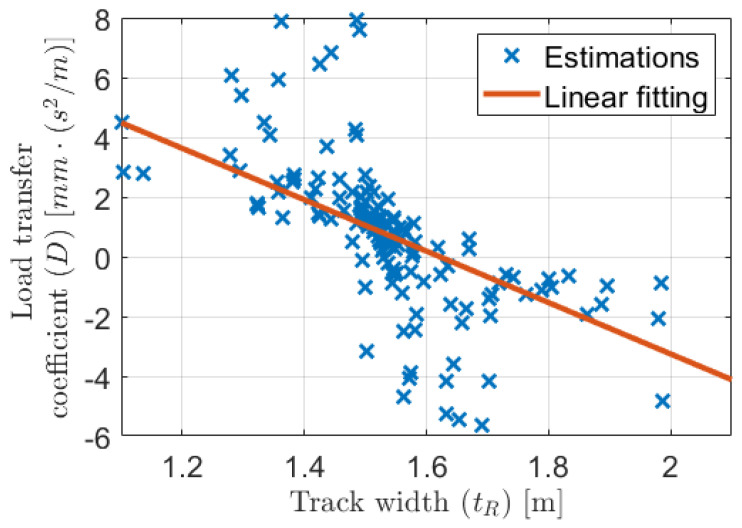
Estimated $t_{R}-D$ values.

Figure 22 shows that the estimated track and load transfer parameters varied significantly in the subtraces, but it was expected. As we can see in the previous sections these parameters uniquely compensated each other to reach optimum calibration on the actual subtrace. Thus, the variation of these two parameters was not random. It can be illustrated well if the load transfer coefficient is plotted as a function of the track, which is presented in Figure 23.

Even though some outliers appeared, the relation between the two parameters was obvious. Consequently, the large variety of the parameters was not an unfavorable and completely noisy phenomenon.The value of the estimated load transfer coefficient was only $\bar{D}=0.7226\;mm\cdot s^{2}/m$, which for example with 3 m/s${}^{2}$ lateral acceleration resulted in 4 mm actual difference between the wheel circumferences. However, in this wheel odometry model, the angular velocity was calculated as the difference of the rear-wheel velocities and these velocities were the product of the wheel rotation and circumference. Consequently, the few millimeters difference could influence the localization significantly, as it was presented in the motivation example in Section 2.3.

Moreover, the noisy estimation was not a problem, if there was enough value to calculate a stable mean. The presented measurement required 23 km to obtain 144 valid estimation points. These resulted in the optimal calibration setting, such as

$$\begin{array}{cccc} \bar{c_{e,RL}} & =c_{e,RL,opt}=1.9503\;m & \sigma_{c_{e,RL,opt}} & =0.0064\;m\\ \bar{c_{d}} & =c_{d,opt}=2.0510\;\mathrm{mm} & \sigma_{c_{d,opt}} & =0.4925\;\mathrm{mm}\\ \bar{t_{R}} & =t_{R,opt}=1.5428\;m & \sigma_{t_{R,opt}} & =0.1486\;m\\ \bar{D} & =D_{opt}=0.7226\;\mathrm{mm}\cdot s^{2}/m & \sigma_{D_{opt}} & =2.6326\;\mathrm{mm}\cdot s^{2}/m \end{array}$$

In parallel with the mean, the standard deviations were also calculated. These are essential when the calibrated odometry model is utilized in a fusion algorithm because the process noise (for example the *P* in a Kalman-filtering) can be estimated easily using the parameter uncertainties.

### 6.3. Validation and Test

The direct validation of the model calibration is difficult due to the true value of these parameters are unknown. Indirectly the calibration performance can be validated by testing the odometry model without any fusion on various subtraces and examine the localization accuracy. The pose error was calculated from the reference measurements. The validation of a model calibration is relevant only if the model is tested in different cases from the ones on which the estimation is executed. However, from the 23 km long measurement only that cases, where the angular velocity was significant, were applied. Furthermore, a new subtrace generation was fulfilled. 400 m long subtraces were generated with 1 s shift between the segments throughout the whole measurement without any subtrace elimination. With this generation, the effect of segments resulting in peak positioning errors was reduced.

The average of the mean position errors with the calibrated odometry model was 4.04 m while the average orientation error was $1.58^{\circ }$. In relative terms, the positioning error was only 1%. If the estimated sideslip was not applied, the error increased but merely to 1.1% which is an excellent result in 400 m long driving using only the wheel encoder measurements and the lateral acceleration signal from the IMU.

The localization performance of the GN-KF calibration is compared with other cases which can be found in Table 4. With the presented cases the whole calibration is executed and the models with the estimated parameters are tested. The necessity of calibration is shown by the fact that the nominal setting resulted in five times higher errors. The impact of the integrated Kalman-filtering is also illustrated, because if the calibration was executed without the KF, the estimated $t_{R}$ and *D* parameters significantly differed from the GN-KF ones, and in parallel, the errors were 2 times higher. Finally, the calibration was performed with the ordinary wheel odometry model as well without lateral dynamics ($\beta_{k}=0$) and neglecting the proposed dynamic wheel model (D=0). The results show that the wheel circumference parameters could be estimated well, but the track estimation was biased. Moreover, the $t_{R}$ parameter moved to the opposite direction from the nominal setting, than in the case of the calibration with the proposed model. Consequently, the average errors were also higher. Therefore, without our novel wheel odometry model or with the neglect of the integrated KF from the loop, only biased track calibration could be performed which decreased the localization performance.

The impact of the dynamic wheel assumption is also illustrated clearly with the examination of the $t_{R}-D$ parameters in the three calibration cases. When the wheel was modeled as dynamic the two $t_{R}-D$ combination determined a straight in the parameter field. This line is almost the same as the linear fitting presented in Figure 23. Furthermore, the ordinary model with static circumferences (only $c_{e,RL}$, $c_{d}$ and $t_{R}$ are the parameters) should be the case with D=0 value. Because the ordinary model fits well to this line at the D=0 point, the $\pm D\cdot a_{y}$ relation should be a proper description of the effect of dynamic load transfer.

It was mentioned in the introduction that the well-calibrated odometry would have several advantages, such as fusion with other cost-effective sensors, or used to calculate other sensor biases. Thus, the calibrated model with the proposed algorithm was tested with different integration times and examined as a motion sensor. The values and the average pose errors can be found in Table 5. The 1% relative position error related to the path length was certainly true from 1 s up to 60 s integration time, which means that the drift of the calibrated odometry was linear in the driven distance.

Calculating the gradient of this drift, the odometry model corresponded to an angular velocity sensor with 0.0024 rad/s, and a speed sensor with 0.07 m/s unknown bias. It is difficult to compare with an accelerometer due to that sensor had quadratic drift in distance, but the 5.96 m error in a 60 s corresponded to 0.0066 m/s${}^{2}$ bias, the 2.23 m in 30 s to 0.0099 m/s${}^{2}$ while the 0.33 m in 5 s to 0.0528 m/s${}^{2}$, at 9 m/s average speed. The average was 0.03 m/s${}^{2}$ unknown bias which with the 0.0024 rad/s angular velocity uncertainty certainly exceeded the accuracy of an automotive-grade IMU. Therefore, the proposed calibrated odometry can be a proper choice to fuse with absolute sensors, such as GNSS and compass to result in an accurate, but still cost-effective localization system.

## 7. Conclusions

In this paper, a novel odometry model, with the integration of dynamic wheel model and lateral dynamics, and a calibration architecture has been presented to improve the localization performance of a self-driving car. In the design, the general requirements of the automotive industry are taken into consideration, thus only cost-effective sensors are used. A unique estimation algorithm of the applied sideslip is also developed in which the key idea is a determination of the zero-crossings of the signal. Due to the nonlinear model behavior, the iterative Gauss–Newton regression is applied for the calibration. The dynamic model estimation requires state initialization, which uncertainty corrupts the calibration. Our proposed method to mitigate this effect is a Kalman-filtering inside the optimization loop. The main contribution is that the precise calibration of the wheel odometry model can be executed only with the dynamic wheel assumption and the integrated filtering in the estimation loop. The method is tested with real experiments, where only automotive-grade onboard GNSS, IMU, and wheel encoder signals are utilized. The results show that with the calibrated model the pose errors are five times lower. Therefore, the proposed odometry model can be an accurate motion estimation sensor and still operates with signals from cost-effective equipment.

The limitation of the method is that though the integrated filtering can reduce the effect of initial state uncertainty, some estimated parameters have high variance. Thus, the proper calibration can be reached only on a long measurement scenario. As a future challenge, the variance should be decreased by the elimination of the initial state uncertainty. Furthermore, the online version of the calibration will be developed.

## Figures and Tables

**Table 1:** Parameters and errors of the motivation example cases I.

	$c_{e,RL}\left[m\right]$	$c_{d}\left[m\right]$	$t_{R}\left[m\right]$	$D[s^{2}]$	${Err}_{p}\left[m\right]$	${Err}_{\psi }{[}^{\circ }]$
Case 1	2	0	1.6	0	19.14	12.37
Case 2	1.95	0	1.6	0	14.78	9.79
Case 3	2	0.001	1.6	0	14.73	9.55
Case 4	2	0.002	1.6	0	10.33	6.73
Case 5	2	0	1.535	0	14.68	10.01
Case 6	1.95	0.002	1.535	0	2.31	1.99

**Table 2:** Parameters and errors of the motivation example cases II.

	$c_{e,RL}\left[m\right]$	$c_{d}\left[mm\right]$	$t_{R}\left[m\right]$	$D[mm\cdot \left(s^{2}/m\right)]$	${Err}_{p}\left[m\right]$	${Err}_{\psi }{[}^{\circ }]$
Case 7: $\beta_{k}=0$	1.9603	1.3800	1.6396	0	2.08	0.51
Case 8: $\beta_{k}\neq 0$	1.9540	1.3360	1.6386	0	1.27	0.43
Case 9: $\beta_{k}=0$	1.9564	2.4752	1.5223	0.9476	1.99	0.52
Case 10: $\beta_{k}\neq 0$	1.9506	2.1173	1.5363	0.6635	1.21	0.42

**Table 3:** Parameters and errors with various $w_{\tilde{\psi }}$ settings.

	$c_{e,RL}\;\left[m\right]$	$c_{d}\;\left[mm\right]$	$t_{R}\;\left[m\right]$	$D\;[mm\cdot \left(s^{2}/m\right)]$	$E_{rr,pos}\;\left[m\right]$	$E_{rr,ori}\;{[}^{\circ }]$
$w_{\tilde{\psi }}=50$	1.9484	2.3130	1.4523	2.5843	0.3472	0.3268
$w_{\tilde{\psi }}=100$	1.9502	2.3065	1.5001	1.3290	0.4253	0.2474
$w_{\tilde{\psi }}=200$	1.9506	2.2448	1.5353	0.8547	0.4715	0.2080
$w_{\tilde{\psi }}=400$	1.9508	2.3011	1.5751	0.4940	0.6165	0.1593
nominal	2.0000	0.0000	1.6000	0.0000	7.0424	4.6946

**Table 4:** Estimated parameters and average test errors.

	$c_{e,RL}\;\left[m\right]$	$c_{d}\;\left[mm\right]$	$t_{R}\;\left[m\right]$	$D\;[mm\cdot \left(s^{2}/m\right)]$	$\bar{E_{rr,pos}}\;\left[m\right]$	$\bar{E_{rr,ori}}\;{[}^{\circ }]$
calibration with GN-KF	1.9503	2.0510	1.5428	0.7226	4.0355	1.5836
nominal setting	2.0000	-	1.6000	-	19.4930	0.8360
calibration without KF	1.9494	2.0821	1.6941	–0.8965	7.8524	3.3390
calibration with ordinary model	1.9479	2.1054	1.6292	-	6.1807	2.6498

**Table 5:** Average pose errors with different integration time.

Integration Time [s]	60	45	30	20	10	5	1
Average subtrace length [m]	550	412	275	183	92	46	9
Average position error $\bar{E_{err,pos}}$ [m]	5.96	4.05	2.23	1.38	0.64	0.33	0.07
Average orientation error $\bar{E_{err,ori}}$ [${}^{\circ }$]	1.93	1.58	1.21	0.93	0.58	0.37	0.14
